# Breeding and Engineering Trees to Accumulate High Levels of Terpene Metabolites for Plant Defense and Renewable Chemicals

**DOI:** 10.3389/fpls.2018.01672

**Published:** 2018-11-20

**Authors:** Gary F. Peter

**Affiliations:** School of Forest Resources and Conservation, Genetics Institute, University of Florida, Gainesville, FL, United States

**Keywords:** resin duct, terpene, conifer, oil gland, *Eucalyptus*, defense, biosynthesis, cell specialization

## Abstract

Plants evolved the capacity to synthesize highly diverse sets of secondary metabolites which are important for plant adaptation and health. In forest trees, many classes of compounds, particularly ones related to defense against insects, fungi, and bacteria accumulate to levels that enable their recovery and commercial use. One of the oldest examples is conifer terpenes, but terpenes are important secondary products from other tree species including eucalypts. Because terpenes, latex, and natural gums are synthesized and stored in specialized secretory glands, ducts, and laticifers in mostly pure forms they can be collected from live trees in addition to being extracted during industrial processing of wood. This minireview describes the potential of breeding and genetic engineering approaches to increase the quantities of terpene secondary metabolites to increase the amount of secondary products and thereby increasing the value of planted and managed forest trees. I advance the view that breeding and genetic engineering of metabolic pathways and specialized cell secretory structures can dramatically increase tissue terpene content.

## Introduction

Plants evolved the ability to synthesize a large diversity of carbon rich compounds, including fatty acids, lipids, phenolics, sterols, tannins, and terpenes. Some of these hydrocarbons can accumulate to high percentages of dry mass. For example, oil seeds accumulate 10–75% triacylglycerol by dry mass, depending on the species. With the goal of producing hydrocarbons directly in plants for renewable chemicals and biofuels, tobacco was engineered using a push-pull-protect strategy to accumulate 15% of leaf dry mass as triacylglycerol (Vanhercke et al., 2014). In addition to hydrocarbons synthesized via primary metabolic pathways, there are many synthesized via secondary metabolic pathways. One of the most diverse sets of plant hydrocarbons are terpenes. Plant terpenes are synthesized via the plastid localized 2-C-methyl-D-erythritol 4-phosphate (MEP) pathway and the cytosolic mevalonate pathway (MVA) (Kirby and Keasling, 2009; Vranova et al., 2013). Terpenes are critical components of plant defense against herbivores, insects, pathogenic fungi, and bacteria (Franceschi et al., 2005). For terpene defenses to be effective, sufficient amounts need to accumulate prior to invasion; however, at these levels they are phytotoxic. To minimize this toxicity, plants evolved the ability to sequester them in extracellular spaces formed during specialized differentiation of secretory tissues (Fahn, 1979, 1988). In a wide variety of plant species terpenes accumulate in extracellular cavities, or lumen that form during differentiation of glandular trichomes (Glas et al., 2012; Huchelmann et al., 2017), secretory cavities, or oil glands (OG) (Mewalal et al., 2017), and resin ducts (Zulak and Bohlmann, 2010). These tissues are specialized for the biosynthesis and storage of terpenes.

In many forest tree species, terpenes are vital chemical, and physical defense compounds, and specialized tissues are crucial to their biosynthesis and storage at high concentrations. For example, in *Eucalyptus* species mono- and sesquiterpenes are important for defense against insects, and herbivores, and are synthesized, and accumulate in secretory cavities, or OG within leaves (King et al., 2006; Naidoo et al., 2014). Eucalypt leaf terpene content ranges from 0.5 to 8% of dry mass and they are commercially extracted and used in the fragrance and pharmaceutical industries and have potential as biofuels (Mewalal et al., 2017). Conifer terpenes are a primary component of a durable, quantitative defense mechanism against stem boring insects and fungal pathogens (Trapp and Croteau, 2001; Franceschi et al., 2005). Constitutive and induced terpenes are critical for resistance (Hodges et al., 1985; Strom et al., 2002; Klepzig et al., 2005). Upon wounding, preformed constitutively produced terpenes are released at the wound site and the insects, and fungi are contained and growth is inhibited. The toxic terpenes cannot only kill the attacking organisms but they also flush and seal the wound site forming a physical barrier protecting the stem from further infection (Trapp and Croteau, 2001). Conifer terpenes are synthesized and accumulate in primary and secondary resin ducts, and form a viscous oleoresin secretion consisting of a complex mixture of volatile monoterpene olefins, and non-volatile diterpenoid resin acids that naturally accumulate to between 2 and 40% of the dry mass of wood (Trapp and Croteau, 2001).

The native levels of terpenes in eucalypt leaves and wood of conifer species is adequate for commercial extraction and use as renewable chemicals, and biofuels. In conifers, commercial scale collection of terpenes occurs today from live pine trees by tapping, solvent extraction of stumps, and during chemical pulping of wood, with global production from all sources about 3.5–4.0 millon tons y^-1^. Southeastern United States pulp mills recover ∼900,000 tons y^-1^ of pine terpenes and fatty acids as crude tall oil (CTO) supporting specialty chemical biorefineries that compete in markets with petroleum derived feedstocks justifying the concept that biofuels from pine hydrocarbons could be profitable without subsidy if supply was increased (American, 2011). In the European Union, CTO is converted to renewable diesel fuel at two industrial scale biorefineries: one with a capacity of 120 million liters y^-1^ (UPM-Kymmene, 2018). Pine monoterpenes can be efficiently dimerized to make jet fuel (Harvey et al., 2010; Mewalal et al., 2017). Globally, pine chemicals are the largest non-food based renewable hydrocarbon supply. However, the pine terpene supply is limited by the relatively low 2–4% average wood terpene content of dry wood.

Here, I discuss three synergistic approaches to increase tissue terpene content that can lead to forest trees with improved resistance to herbivores, insects, and pathogens as well as greater supplies of terpenes for renewable chemicals, and biofuels. Three main approaches to increase tissue terpene content considered are: (1) increase the number of specialized secretory glands and duct in plant organs to create greater biosynthetic, and storage capacity; (2) increase luminal storage volume of specialized secretory tissues; and (3) enhance carbon flux into terpene biosynthetic pathways.

## Increase Secretory Tissues in Plant Organs

**FIGURE 1:**
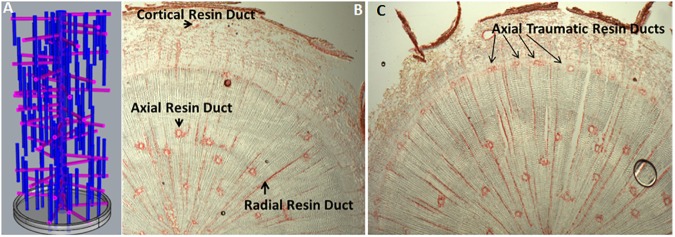
Resin duct organization and induction. **(A)** A theoretical three dimensional model depicting the interconnectivity of constitutive axial and radial resin ducts in woody stems of Pinaceae; **(B)** Transverse cryosection of an untreated 1-year-old loblolly pine stem, stained with safranin red, showing constitutive axial and radial resin ducts in the wood and cortical resin duct in the bark (Peter, 2018, Unpublished); **(C)** Transverse cryosection of 1-year-old loblolly pine stem 10 days after treatment with 10 mM methyl jasmonate (Martin et al., 2002), stained with safranin red showing newly formed axial traumatic resin ducts in the cambial zone/young xylem (Peter, 2018, Unpublished).

**FIGURE 2:**
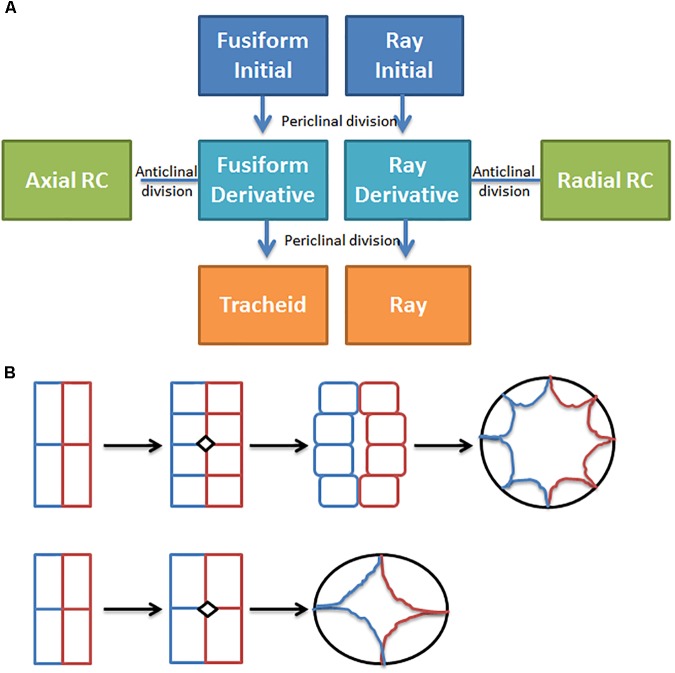
Resin duct formation in the cambial zone. **(A)** Simple conceptual model for secondary resin duct formation; **(B)** Transverse plane drawing of two ways cambial zone cells may divide to form epithelial cells in more narrow or wider axial resin ducts, adapted from Sato and Ishida (1983), the colors indicate cell lineage.

In conifers, primary resin ducts (RD) form in needles and non-woody shoots, and secondary RD form in the secondary xylem, or wood (Larson, 1994). Although there is limited knowledge of primary RD formation, secondary RD formation, and function are better studied (Larson, 1994; Zulak and Bohlmann, 2010). Within a network of secondary RDs, terpenes are synthesized by heterotrophic epithelial cells that line the duct and are secreted into the lumen where they accumulate (Fahn, 1979; Trapp and Croteau, 2001; Zulak and Bohlmann, 2010; Figure 1). The lumens of axial and radial RDs are interconnected such that upon wounding the terpene oleoresin can flow from within the wood toward the surface of the stem (Larson, 1994; Figure 1A). The interconnectedness of the network is also important for the RD to obtain sucrose and nutrients from the phloem at the periphery of the stem through the live ray cell files. During stem diameter growth, axial, and radial RDs differentiate in the cambial zone from derivatives of fusiform and ray initials (Larson, 1994). Two adjacent derivatives begin dividing anticlinally to form a multicellular pre-duct structure which subsequently form the lumen schizogenously by separation of the cells at the middle lamella (Larson, 1994; Figure 2).

The genetic mechanisms that control constitutive RD cell fate and differentiation are not known. In loblolly pine, we showed that constitutive axial resin duct number (Westbrook et al., 2015) is under moderate genetic control, inherited as a polygenic trait, positively genetically correlated with xylem growth, and has sufficient variation for breeding to increase constitutive axial RD number about 10% in a single generation. *Picea* species (spruce) have emerged as the best-studied experimental system for dissecting the developmental signals and biochemical basis of induced terpene resin formation (Franceschi et al., 2005; Keeling and Bohlmann, 2006; Zulak and Bohlmann, 2010). Insect feeding, wounding, ethylene, and methyl jasmonate (MeJ) treatments induce development of traumatic resin ducts (TRD), which develop as a single contiguous row of elements derived from xylem mother cells in the cambial zone (Franceschi et al., 2005). Anatomical time course studies show that axial TRD take about 9–36 days to differentiate depending on the species (Larson, 1994; Nagy et al., 2000). In Norway spruce, a detailed time course of TRD formation and terpene synthesis in wood after MeJ application shows that geranylgeranyl-pyrophosphate synthase, monoterpene, and diterpene synthase activity began increasing after 5 days and peaked ∼15 days and increases in wood monoterpene and diterpenoid content were observed after 10 days and peaked between 15 and 25 days (Martin et al., 2002). New TRD are observed at 10 days and supports the conclusion that in spruce wood newly synthesized terpene is only present in the newly formed TRD and not in undifferentiated ray cells. Thus, increasing RD numbers should be an effective strategy to increase wood terpene content. Exogenous application of MeJ induces axial TRD in the cambial region (Martin et al., 2002; Figure 1C), and application of inhibitors of ethylene biosynthesis suppresses jasmonate TRD induction, suggesting that jasmonate induces ethylene synthesis which then induces TRD formation (Hudgins and Franceschi, 2004). Consistent with this model, MeJ application to sitka spruce bark rapidly upregulates genes that code for the rate limiting enzyme for ethylene biosynthesis, 1-aminocyclopropane carboxylic acid synthase (Ralph et al., 2007). These observations support that genetically increasing ethylene, or jasmonate biosynthesis, or signaling in the cambial zone should increase RD number and lead to greater wood terpene content.

To identify RD specific and jasmonate regulated genes in RDs, the transcriptomes of isolated cortical RDs from bark of Norway spruce were compared before and after treatment with MeJ (Celedon et al., 2017). In untreated bark, 863 cortical RD specific transcripts (Tau score > 0.75) were identified, and in MeJ treated bark, 766 cortical RD specific transcripts were identified. Of these 766 transcripts, about 648 were also identified as being specific to untreated cortical RD, indicating that 85% of the cortical RD specific genes remain RD specific 8 days after MeJ treatment (Celedon et al., 2017). As expected in the untreated cortical RD, specific transcripts included genes that code for all MEP biosynthetic enzymes, and a large number of cytochrome P450s, and terpene synthases. In addition, they included 34 transcripts that are annotated as transcription factors or encode putative DNA binding domains. There is potential that these regulators mediate RD specification and terpene pathway gene expression. In bark, ∼3,700 genes were differentially expressed 8 days after MeJ treatment; however, the number of jasmonate responsive genes in cortical RD was not reported. The terpene pathway genes in cortical RD showed that they were reduced 8 days after MeJ treatment relative to controls (Celedon et al., 2017). Overall, this data shows that in control and jasmonate treated bark, the genes coding for terpene biosynthesis are specifically expressed in cortical RDs.

Manipulating ethylene or jasmonate may induce more secondary RDs but could also have unintended effects on tracheids, so targeted genetic manipulation may be preferred. Targeted manipulation is limited by our lack of knowledge of the molecular mechanisms that regulate resin duct cell fate, differentiation, and function. Figure 2 shows a simple conceptual model for secondary axial and radial RD formation. During normal stem diameter growth, constitutively formed secondary axial RDs appear to occur randomly throughout the wood volume but are typically adjacent to one or more ray cell files (Figure 1). While, derivatives of fusiform initials typically differentiate into tracheids, those adjacent to ray initials can also differentiate into axial resin ducts. Because the vast majority of fusiform derivatives differentiate into tracheids, for axial RDs to form, a first step is to inhibit regulators that specify tracheid cell fate. Although the molecular control of tracheid cell specification is presently unknown in conifers, vascular related NAC domain transcription factors function as master switches in angiosperms xylem (Zhang et al., 2014; Jokipii-Lukkari et al., 2017). In analogy to xylem cell specification, it is reasonable to hypothesize that a master transcriptional regulator specifies RD identity. Based on parsimony, this regulator also will be responsive to ethylene and/or jasmonate, and therefore these hormones could be important for constitutive RD formation in addition to TRD formation. However, the overlap between the traumatic RD and constitutive RD pathways is not known. It is also unknown whether the fusiform derivatives need to first have ray cell identity to differentiate into RDs or whether derivatives can directly acquire RD cell fate. Constitutive radial RDs also form in the cambial zone; however, it is unclear if they can differentiate within existing ray cell files deeper in the stem. Once a radial RD forms, typically next to an axial RD, the radial RD fate extends within that ray cell file out to the cambial meristem (Figure 1). To discover genes involved in RD formation, we are using association genetics in loblolly pine. With single nucleotide polymorphisms (SNP) in 4027 genes we identified 16 well supported candidate genes associated with axial RD number, some of which were also significant for oleoresin flow (Westbrook et al., 2015). More recently, we genotyped ∼70,000 SNPs in this population and identified significant SNPs in an additional 18 genes for constitutive axial RD number (Peter, 2018, Unpublished). We are investigating the importance of some of these candidate genes in RD specification through genetic engineering.

## Increase Luminal Volume of Secretory Tissues

Conifer species differ in the organization of terpene resin producing cells and tissues. Abies species form both resin cells and multicellular resin blisters, while some Pinaceae species form constricted resin passages and other Pinaceae species form non-occluded resin ducts (Penhallow, 1907; Lewinsohn et al., 1991). Although no rigorous comparison amongst these species is available to separate the significance of resin structure number and extracellular volume, in general, wood terpene content is greater in species with resin ducts compared with species with resin cells (Lewinsohn et al., 1991).

Increasing luminal volume in addition to increasing RD number should lead to wood with greater terpene content. What controls RD luminal diameter is unknown, but lumen volume tends to be greater in RDs that form from a greater number of early cell divisions during differentiation (Figure 2B). Possibly four instead of two cambial derivatives could be induced to divide and commit to RD large diameter lumen would form. In loblolly pine, the length of axial RD range from 5 to 20 cm and average 10.4 cm, and in slash pine average RD length was more than double (49.8 cm vs. 20 cm) at 20 versus 10 years (Larson, 1994). In southern pines, axial RDs are 10–100 times longer than individual fusiform initials (3–5 mm) suggesting that a large number of contiguous fusiform initial derivatives within a single file need to commit to form axial RDs. The mechanism(s) which mediates axial RD length are unknown, but I hypothesize that cell to cell communication signals that specify RD identity are important for coordinating differentiation of the 10–100s of cambial meristem derivatives. If this hypothesis is correct, then discovery of this transmissible signal could enable engineering of larger RD and thereby increase wood terpene content. In *Eucalyptus polybractea*, the number of OG is correlated well with leaf terpene content (King et al., 2006; Goodger and Woodrow, 2012). Analysis of individual OG shows they have very similar capacities, suggesting that the storage volume of the cavity is relatively tightly controlled, and it was hypothesized that gland capacity is controlled by leaf expansion (King et al., 2006). If King’s hypothesis is correct, then increasing leaf thickness could increase cavity volume. Because leaf terpene content is limited not only by the number of OG but also by luminal volume of the gland it could be possible to increase this volume by engineering OG to be more duct like, similar to primary RD in conifers. I hypothesize that the difference between formation of spherical glands and elongated ducts is related to the existence of a cell to cell signaling mechanism which evolved in Pinaceae that mediates duct formation.

## Enhance Carbon Flux to Terpenes

Plants synthesize monoterpenes and diterpenes via the plastid localized 2-C-MEP pathway, and sesquiterpenes, and triterpenes via the cytosolic MVA (Kirby and Keasling, 2009; Vranova et al., 2013; Banerjee and Sharkey, 2014). The MEP pathway is considered the high flux pathway in part because many species including *Eucalyptus* are rich in monoterpenes and conifers are rich in monoterpenes, and diterpenoids. The first committed step in the MEP pathway is catalyzed by 1-deoxyxyulose 5-phosphate synthase (DXS). Constitutive overexpression of DXS enzymes elevated terpene alkaloids in *C. roseus* (Peebles et al., 2011), taxadiene in *Arabidopsis thaliana* expressing a taxadiene synthase (Botella-Pavia et al., 2004), and terpenes 2–3.5 fold in leaves of *L. latifolia* (Munoz-Bertomeu et al., 2006). DXS is sensitive to feedback inhibition by isoprenoid precursor’s isopentenyl diphosphate and dimethylallyl diphosphate which can control carbon flux through the pathway (Banerjee et al., 2013; Banerjee and Sharkey, 2014). A mutant form of poplar DXS was described that is less sensitive to feedback regulation; however, this has yet to be tested in plants (Banerjee et al., 2016). In spruce, the DXS gene family codes for type I and type II enzymes. Type II DXS genes are involved in secondary metabolism and in spruce are upregulated by wounding and fungal infection, suggesting that DXS overexpression should enhance wood terpene synthesis (Phillips et al., 2007). Upregulation of deoxyxyulose 5-phospate reductoisomerase (DXR) leads to a 50% increase in terpene based essential oils stored in globular trichomes of peppermint (Mahmoud and Croteau, 2001), and increased isoprene products, e.g., chlorophyll, β-carotene and taxadiene in *A. thaliana* expressing taxadiene synthase (Carretero-Paulet et al., 2006).

The plant MEP pathway is conserved with the deoxy-xyulose 5-phosphate (DXP) pathway in microbes which has been improved through extensive genetic engineering (Ajikumar et al., 2008; Wang et al., 2009; Kirby et al., 2016) and synthetic biology approaches (Keasling, 2014). The results from many of these studies show that larger increases in isoprene products occur when multiple genes are manipulated in combination (Ajikumar et al., 2008; Wang et al., 2009). Changes to multiple genes in plants will require advances in genetic engineering efficiencies and more effective gene editing tools, particularly for conifers which have very large genomes and large gene families (Birol et al., 2013; Nystedt et al., 2013; Neale et al., 2014; Wegrzyn et al., 2014; Zimin et al., 2014). A particularly interesting finding was the identification of two *Escherichia coli* genes that can bypass the DXS catalyzed step (Kirby et al., 2015). By-passing DXS should improve carbon efficiency as DXS releases CO_2_ when it condenses pyruvate and glyceraldehyde 3-phosphate into the five carbon 1-DXP product. The wild type yajO and mutant forms of ribB gene product were shown to convert ribulose 5-phosphate into DXP *in vitro* and upon expression in *E. coli* improve terpene synthesis (Kirby et al., 2015). Transgenic loblolly pine constitutively expressing plastid localized YajO and RibB(G108S) showed significantly increased terpenes in the wood of 1-year-old seedlings (Kirby et al., 2018, Unpublished).

## Conclusion

There is great opportunity to increase terpene hydrocarbon content in eucalypts and conifers through breeding and genetic engineering. Continued discovery of the molecular mechanisms that control OG and RD specification and cell to cell signaling have the potential to enable genetic engineering and targeted breeding to increase the number of resin ducts and OG and their size leading to trees with greater levels of constitutively produced terpenes. Capitalizing on the advances in genetic engineering and synthetic biology with the MEP pathway in microbes should further enable greater carbon flux and carbon efficiency.

## Author Contributions

The author confirms being the sole contributor of this work and has approved it for publication.

## Conflict of Interest Statement

The author declares that the research was conducted in the absence of any commercial or financial relationships that could be construed as a potential conflict of interest.
